# Elevated NF-κB/SHh/GLI1 Signature Denotes a Worse Prognosis and Represent a Novel Potential Therapeutic Target in Advanced Prostate Cancer

**DOI:** 10.3390/cells11132118

**Published:** 2022-07-05

**Authors:** Davide Vecchiotti, Daniela Verzella, Mauro Di Vito Nolfi, Daniel D’Andrea, Irene Flati, Barbara Di Francesco, Jessica Cornice, Edoardo Alesse, Daria Capece, Francesca Zazzeroni

**Affiliations:** 1 Department of Biotechnological and Applied Clinical Sciences (DISCAB), University of L’Aquila, 67100 L’Aquila, Italy; davide.vecchiotti@univaq.it (D.V.); daniela.verzella@univaq.it (D.V.); mauro.divitonolfi@univaq.it (M.D.V.N.); irene.flati@graduate.univaq.it (I.F.); barbara.difrancesco@univaq.it (B.D.F.); jessica.cornice@graduate.univaq.it (J.C.); edoardo.alesse@univaq.it (E.A.); francesca.zazzeroni@univaq.it (F.Z.); 2 Interdisciplinary Biomedical Research Centre, College of Science and Technology, Nottingham Trent University, Clifton NG11 8NS, UK; daniel.dandrea@ntu.ac.uk

**Keywords:** prostate cancer, NF-κB, Sonic Hedgehog, GLI1

## Abstract

Prostate cancer (PCa) is the second most frequent cancer in men worldwide. NF-κB seems to play a key role in cell survival, proliferation and invasion, sustaining the heterogeneous multifocal nature of PCa. In recent years, the Hedgehog (Hh) signaling pathway has attracted attention as a therapeutic target due to its implication in tumorigenesis and metastasis in several types of cancer, including PCa. Although it is well-known that Sonic Hedgehog (SHh) is a transcriptional target of NF-κB(p65), and that GLI1 is the effector of this crosstalk, the precise role played by this axis in PCa is still not completely clear. Here, we set out to explore the correlation between NF-κB activation and SHh pathways in PCa, investigating if the interplay between NF-κB(p65) and SHh-GLI1 in advanced PCa could be a prospective therapeutic target. Our findings demonstrate that a *NF-κB-SHh-GLI1* gene signature is enriched in PCa patients featuring a higher Gleason score. Moreover, elevated levels of this signature are associated with worse prognosis, thus suggesting that this axis could provide a route to treat aggressive PCa.

## 1. Introduction

In 2020, the global cancer burden reached a total of 19.3 million new cases and 10.0 million deaths. In this context, prostate cancer (PCa) represents the second most frequent cancer in men, accounting for 1.4 million (7.3%) of newly diagnosed cases and being in the top five most diagnosed neoplasms [1]. As observed in other cancers, the accumulation of many somatic genetic and epigenetic changes sustains prostate tumorigenesis and progression, but the molecular basis of this disease is still unsolved [2]. The epithelial compartment is widely recognized as the site of origin for PCa, although it is not clear which is the specific cell subtype from which the tumor arises. PCa progresses from an in situ, curable, androgen-dependent (AD) disease to a highly invasive, metastatic, and androgen-independent (AI) malignancy, also known as castration resistant prostate cancer (CRPC) [3,4]. A combination of PSA levels, Gleason score and TNM stage may be used nowadays to stratify patient disease risk and help physicians to choose the most appropriate therapeutic strategy [5,6]. Due to the long-life expectancy, low-risk AD-PCa patients are candidates for active surveillance (AS) watchful waiting to monitor potential cancer progression, while curative treatment options such as hormone therapy, radical prostatectomy and radiotherapy are the gold standard approaches for intermediate and high-risk AI-patients [7].

Within the complex molecular maze underlying PCa, key roles in cell survival, promotion, proliferation and invasion seem to be played by NF-κB(p65) [8,9,10]. The NF-κB transcription factor proteins are pivotal regulators of cell growth, differentiation, apoptosis [11] immune responses, inflammation and tumor initiation and progression, both in vertebrates and invertebrates [12,13]. The NF-κB family includes five transcription factor proteins, NF-κB1 (p50/p105), NF-κB2 (p52/p100), RelA (p65), RelB and c-Rel, which can dimerize to form all possible combinations of homo and heterodimers. The prototypical form of NF-κB is the heterodimeric p50/p65 complex [14]. *In vitro* and *in vivo* studies support an active role for NF-κB(p65) in PCa, where constitutive NF-κB(p65) overexpression has been related to a higher Gleason score and a poorer prognosis [15,16,17,18,19,20].

In recent years, due to its implication in tumorigenesis and metastasis, the Hedgehog (Hh) signaling pathway has attracted the attention of both pharma and academia as a promising therapeutic target [21,22,23,24,25]. Originally discovered in Drosophila, Hh was later recognized as an evolutionarily conserved pathway in mammals, where the best characterized ligand is Sonic Hedgehog (SHh) [26,27]. SHh ligand binds to its receptor Patched (PTCH) thus activating the Hh signaling pathway and leading to GLI1 transcription factor induction. Upregulation of SHh signaling in PCa tumorigenesis has been demonstrated in several *in vitro* and *in vivo* studies, and correlates with a higher Gleason score, thus underscoring the key role of the SHh pathway in CRPC [28,29,30,31,32]. Given that SHh and NF-κB(p65) control genes that are involved in several altered processes in PCa, and that SHh is a transcriptional target of NF-κB(p65) [33], we investigate the role of the NF-κB(p65)-SHh-GLI1 axis in a specific subset of advanced PCa, with the aim to identify novel molecular targets for future CRPC therapies.

## 2. Material and Methods

### 2.1. Prostate Adenocarcinoma Tissue Samples

Prostate Cancer-Normal Tissue Array (CA3) was purchased by SuperBioChips Tissue Array (Tema Ricerca Srl, Bologna, Italy).

### 2.2. Cell Lines

Human PCa cell lines PC3 and DU145 were cultured in RPMI (Roswell Park Memorial Institute) 1640 and supplemented with 10% FBS (Fetal Bovine Serum) (GIBCO, Carlsbad, CA, USA); LNCaP was cultured in RPMI 1640 and supplemented with 10% FBS, 1% HEPES and 1% NaPy (Sodium Pyruvate). All media were supplemented with L-glutamine 2 mM (GIBCO, Carlsbad, CA, USA) and Penicillin-StreptoMycin 50 U/mL (GIBCO, Carlsbad, CA, USA). TPCA-1 (T1452, Sigma-Aldrich, Merck KGaA, Darmstadt, Germany) and GANT61 (#ALX-270-482-M001, Enzo Lifesciences, New York, NY, USA) inhibitors were used at showed concentration for 72 h alone or in combination. All cell lines were cultivated at 37 °C in 5% CO_2_ humidity and authenticated by STR DNA Profiling Analysis (ATCC).

### 2.3. Lentiviral Production and Infections

The DNA sequences encoding shRNA specific for human p65 (sh-p65: 3′-GCATCCAGACCAACAACAA-5′) and the control non-specific sequences (sh-ns: 3′-CAGTCGCGTTTGCGACTGG-5′) were introduced between the XhoI and HpaI restriction sites of the lentiviral vector pLentiLox3.7 (pLL), expressing eGFP (kindly provided by Prof. Guido Franzoso, Imperial College London, UK). High-titer lentiviral preparations and infection were carried out as previously reported [34].

### 2.4. RNA Extraction and Quantitative Real Time Polymerase-Chain Reaction (qRT-PCR)

Total RNA was extracted from each cell line using Trizol reagent (Cat: 15596026, Invitrogen, Carlsbad, CA, USA) according to the manufacturer’s specifications. RNA (1µg) was reverse transcribed using the GeneAmp^®^RNA PCR Kit (Cat: 10783837, Applied Biosystems, Inc Foster City, CA, USA). qRT-PCRs were carried out using the TaqMan^®^ Universal PCR Master Mix (#4304437, Applied Biosystems) on an ABI 7500 Fast Real-Time PCR machine (Life Technologies). The following TaqMan ^®^ gene Expression Assays were used: GLI1 Hs 00171790_m1 FAM, SHH Hs 00179843_m1 FAM, GAPDH Hs 02786624_g1. Experimental Ct values were normalized to the GAPDH gene and relative mRNA expression was calculated using a reference sample.

### 2.5. Immunohistochemistry

Immunohistochemistry (IHC) was performed on tissue arrays from PCa (CA3, SuperBioChips Tissue Array, Tema Ricerca, Italy) as previously described [35]. The primary antibodies used were as follows: anti-p65 (RB-1638-R7, ready to use, NeoMarker, Thermo Fisher Scientific, Fremont, CA, USA); anti-Shh (sc-1194 N19) (1:50, Santa Cruz Biotechnology); anti-GLI1 (sc-20687 H-300) (1:50, Santa Cruz Biotechnology, Dallas, Texas, USA). Immunoreactions were visualized using the Avidin/Biotinylated enzyme Complex (ABC) (VECTASTAIN Elite ABC System, Burlingame, CA, USA) and sections were counterstained using Mayer’s hematoxylin (Emallume Carazzi Bio-Optica, Milano, Italy). Histological images were acquired using a Nikon Eclipse E200 microscope equipped with a Leica DFC310 FX Digital Camera. The percentage of positive area was quantified using the QuPath Software [36].

### 2.6. Western Blot

Western blots were performed using ECL (Amersham). Proteins were extracted using RIPA buffer (1× phosphate buffered saline, 1% NP40, 0.5% sodium deoxycholate, 1% SDS, 0.1 mM PMSF, 1 µg/ml aprotinine, 0.1 M Na_3_VO_4_) supplemented with complete mini EDTA-free protease inhibitors (Roche Molecular Biochemicals, Basel, Switzerland). A total of 30 µg of protein extracts were loaded onto an SDS-PAGE. The antibodies used were as follows: NF-κB p65 (1:1000, sc-372) and Shh (1:500, sc-9024) were purchased from Santa Cruz Biotechnology; Shh (1:1000, #2207), GLI1 (1:1000, #2643), IκBα (1:1000, #9242), and GAPDH (1:1000, #5174) were purchased from Cell Signaling technologies. Densitometric analysis of immunoblotting was performed as reported in [37].

### 2.7. Viability

Cell proliferation was detected using CellTiter 96^®^ Aqueous One Solution Assay (MTS) (G3580, Promega, Madison, WI, USA) and CellTiter-Glo^®^ 3D Cell Viability Assay reagent (G9681, Promega) according to the manufacturer’s instructions. Cells were seeded in 96-well plates at a density of 5000 (PC3 and DU145) or 10,000 (LnCAP) cells/well after counting with a Cyto Smart cell counter (Corning, New York, NY, USA). Cell plates were cultivated at 37 °C at the indicated time points. CellTiter 96^®^ Aqueous One Solution Assay (MTS) was determined by measuring the absorbance at 490 nm using a µ-Quant plate-reader (Bio-Tek Instruments, Winooski, VT, USA), while the CellTiter-Glo^®^ 3D viability assay was determined with a Packard Lumicount Microplate Reader BL10000 at indicated time points. All experiments were performed three times in triplicate.

### 2.8. Evaluation of NF-κB Activity

NF-κB activity was measured in nuclear protein extracts (15 μg) by the TransAM TM NF-κB p65 protein assay (Active Motif, Carlsbad, CA, USA), an ELISA-based method designed to specifically detect and quantify NF-κB p65 binding. The assay was performed according to the manufacturer’s protocol and analyzed by measuring the absorbance at 450 nm using a µ-Quant plate-reader (Bio-Tek Instruments, Winooski, VT, USA).

### 2.9. Luciferase Assay

Cells were transiently transfected with reporter 12xGLI-luc and pGL4.32 [luc2P/NF-κB-RE/Hygro]. The 12xGLI-luc reporter contains twelve copies of an GLI-response element and was a gift from E. De Smaele, see [38] for details. The pGL4.32 [luc2P/NF-κB-RE/Hygro] was obtained from Promega (Cat N: E849A, Promega) contains five copies of an NF-κB response element (NF-κB-RE) that drives transcription of the luciferase reporter gene luc2. Transient transfections were carried out using Fugene (Cat N: E2312, Promega), following the manufacturer’s protocol. A Renilla luciferase expression plasmid, pRT-TK, was co-transfected as an internal control. Cells were collected 48 h after transfection. 

A luciferase activity assay was performed using a dual-luciferase reporter assay system (Cat N: E1910, Promega) following the manufacturer’s protocol and normalized to Renilla luciferase activity. Relative promoter fold activation of the reporter plasmids was calculated as the ratio of relative luciferase activity values from each sample relative to the corresponding internal control. All experiments were performed in triplicate.

### 2.10. Analysis on TCGA Dataset

Expression and survival data for the analysis of The Cancer Genome Atlas (TCGA) Prostatic Cancer (PRAD) program were from the Pan-Cancer Atlas publication [39] and downloaded from the Genomic Data Common repository (https://gdc.cancer.gov/node/905/, accessed on 1 April 2022). Expression values from the same sample but from different vials/portions/analytes/aliquots were averaged. Analyses were restricted to patients who did not receive radiation therapy. The *p65-SHh-GLI1* gene signature expression levels were performed considering the average of the z-score scaled expressions of the genes in the signature. For progression free interval (PFI) analysis, patients were stratified into two groups based on the signature expression levels, using 75th as the threshold. The curves were estimated using the Kaplan–Meier method, and the differences were tested with the log-rank test, using the survival package. *p*-values < 0.05 were considered statistically significant.

### 2.11. Synergism Analysis

Drug combination responses were calculated based on the Bliss model using SynergyFinder 2.0 [40]. Deviations between observed and expected responses with positive and negative values denote synergistic (>10), additive (from −10 to 10) and antagonistic (<−10) responses, respectively. For estimation of outlier measurements, the cNMF algorithm in SynergyFinder 2.0 was utilized [41].

### 2.12. Statistical Analysis

Statistical significance of the TCGA dataset for two-sample comparisons was calculated by a two-tailed Student’s *t*-test (two-sided) or Mann–Whitney U-test (two-sided), depending on the distribution of the data, as indicated. All the statistical analyses were performed with R software (version 4.0.5). Immunohistochemistry quantification, MTS assay and luciferase assay results were expressed as means ± SD. The data were analyzed using GraphPad Prism software and the statistical analysis was performed using the unpaired 2- tailed Student’s *t*-test or Spearman R2 test. The *p*-values less than 0.05 were considered significant.

## 3. Results

### 3.1. NF-κB/SHh/GLI1 Signature Expression Correlates with Poor Clinical Outcome in PCa

**Figure 1:**
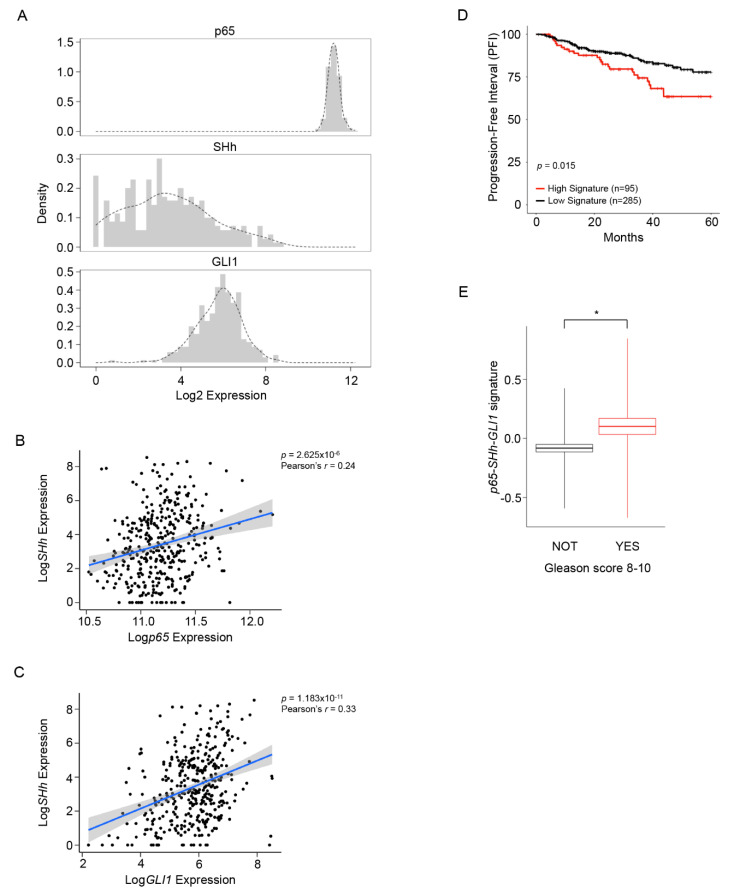
The correlation between the NF-κB-SHh-GLI1 axis and poor clinical outcome in PCa. (**A**) Histogram and sample density (dotted line) plots of *p65*, *SHh* and *GLI1* expression levels in patients with prostate adenocarcinoma from the TCGA dataset (*n* = 380). (**B**,**C**) Shown are the correlations between *p65* and *SHh* mRNA expression (**B**) and *GLI1* and *SHh* mRNA expression (**C**) in patients from (**A**). Pearson’s correlation values and *p*-values are shown. (**D**) Progression Free Interval (PFI) curves in patients from (**A**) stratified based on tumor-associated *p65-SHh-GLI1* signature expression using the 75th percentile as a stratification threshold. (**E**) Box plots showing the expression levels of the *p65-SHh-GLI1* signature in patients with a Gleason score <8 (black, *n* = 252) and ≥8 (red, *n* = 128) from (**A**). Shown in the box plots are the mean (horizontal lines), mean ± SEM (box outlines), or mean ± SD (vertical lines). Statistical significance was calculated by the 2-tailed Student’s *t*-test. * *p* < 0.05.

To better investigate the existing relationship between NF-κB(p65) and SHh-GLI1 within the PCa and if this axis exerted an active role in CRPC, we analyzed a publicly available dataset of 482 PCa patients (Figure 1 and Figure S1). As expected, NF-κB(p65) expression was high and homogeneous in all patients, while SHh and GLI1 showed a more heterogeneous expression within the entire dataset (Figure 1A). We found a significant correlation between *NF-κB(p65)* and *SHh* expression (Pearson’s r = 0.24), as well as between *SHh* and *GLI1* mRNA levels (Pearson’s r = 0.33) (Figure 1B,C). Next, we developed a prognostic gene signature based on *NF-κB(p65)*, *SHh* and *GLI1* expression to stratify patients into low and high signatures. Analysis of the progression free-interval (PFI) at five years demonstrated that elevated expression of the *NF-κB(p65)-SHh-GLI1* signature correlated with a worse prognosis (Figure 1D). Moreover, to further validate the prognostic role of the NF-κB(p65)-SHh-GLI1 axis, we investigated the expression levels of the *NF-κB(p65)-SHh-GLI1* signature in patients with high (8–10) (High risk) and low (6–7) (Low risk) Gleason scores. Our analysis demonstrated that the *NF-κB(p65)-SHh-GLI1* signature is significantly upregulated (*p* = 0.015) in patients with more aggressive (High risk) PCa (Figure 1E).

### 3.2. High NF-κB Activity Strongly Correlates with GLI1 Expression in PCa

**Figure 2:**
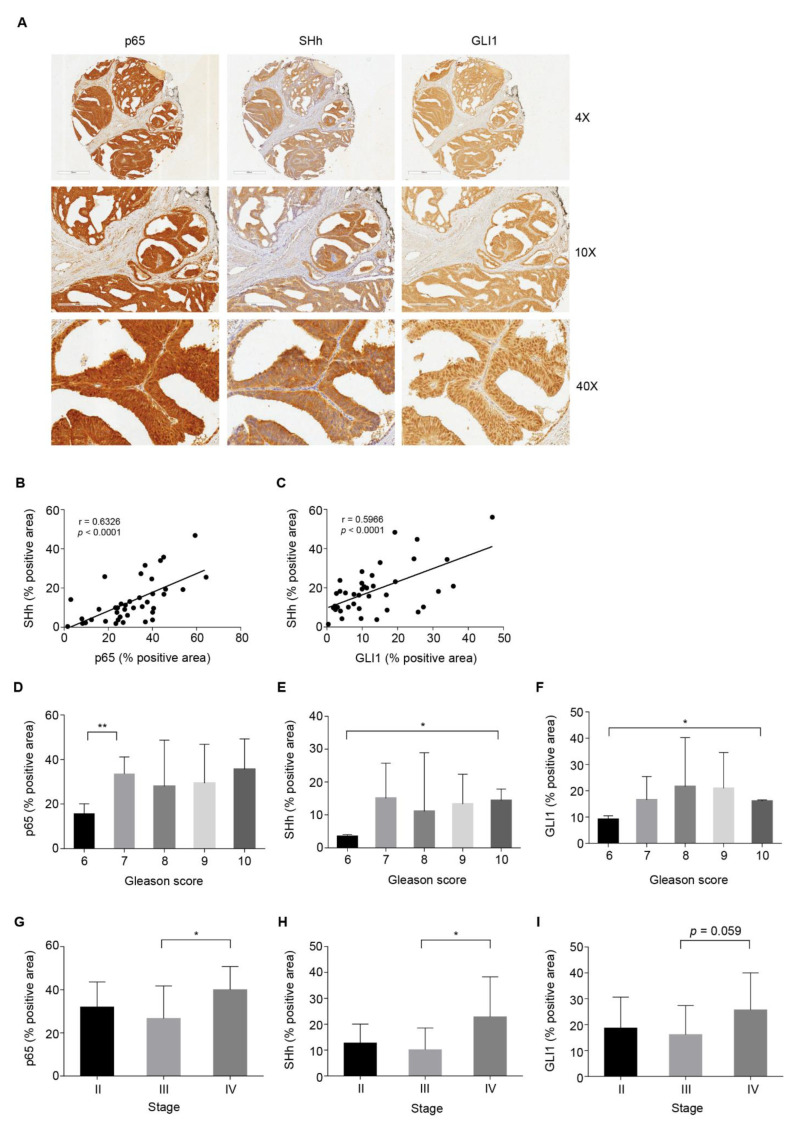
NF-κB-p65 high expression correlates with SHh and GLI1 expression in PCa. (**A**–**I**) IHC analysis for p65, SHh and GLI1 on PCa tissue array (TMA). (**A**) Images of IHC staining of the representative tumor section core. Scale and magnifications are shown. (**B**,**C**) Correlation between the percentage of positive area for p65 and SHh (**B**) and SHh and GLI1 (**C**) in the TMA from (**A**). r, Pearson correlation coefficients. *p* < 0.0001. (**D**–**F**) IHC analysis showing the percentage of positive area for p65 (**D**), SHh (**E)** and GLI1 (**F**) in PCa tissue samples from (**A**) stratified by Gleason Score. (**G**–**I**) IHC analysis showing the percentage of positive area for p65 (**G**), SHh (H) and GLI1 (**I**) in PCa tissue samples from (**A**) stratified by stage of cancer. (**D**–**I**) Gleason score 6, *n* = 2; Gleason score 7, *n* = 15, Gleason score 8, *n* = 6; Gleason score 9, *n* = 15; Gleason score 10, *n* = 2; Stage II, *n* = 7; Stage III, *n* = 23; Stage IV, *n* = 10. Unpaired Student *t*-test or Mann–Whitney U-test were used for statistical analysis. * *p* < 0.05; ** *p* < 0.01.

To evaluate if there was a positive correlation between NF-κB(p65) activation and SHh expression in PCa, a panel of 40 human prostate adenocarcinoma tissues and nine normal prostate tissues spotted on a tissue array were analyzed by IHC. NF-κB(p65), SHh and GLI1 were expressed in all tissues analyzed, showing higher NF-κB(p65) and GLI1 expression levels in tumor vs. normal samples. Indeed, SHh showed the same variability observed in the TGCA analysis (Figure S2C–E; see also Figure 1A). In particular, NF-κB(p65) expression was localized both in the nucleus and into the cytoplasm; SHh expression was mainly cytoplasmic, while GLI1 expression was clearly nuclear (Figure 2A; Figure S2A,B). A significant correlation between NF-κB(p65) activation and the overexpression of SHh or GLI1 was demonstrated (Figure 2B,C). Indeed, Spearman analysis revealed a significantly high correlation between p65 and SHh protein expression (*p* < 0.0001) (Figure 2B) and good co-expression between SHh and GLI1 (*p* < 0.0001) (Figure 2C). In order to investigate the clinical significance of this axis during tumorigenesis, the protein levels of p65, SHh and GLI1 were assessed across PCa samples stratified based on Gleason score and stage. All the three markers analyzed showed higher expression levels in PCa with a higher Gleason score (Figure 2D–F) and stage (Figure 2G–I). These data suggest that in advanced PCa, p65 strongly correlates with SHh and GLI1, confirming a key role for NF-κB(p65) and SHh pathways in this tumor.

### 3.3. NF-κB and SHh Pathways Are Activated in Androgen-Independent PCa Cell Lines

**Figure 3:**
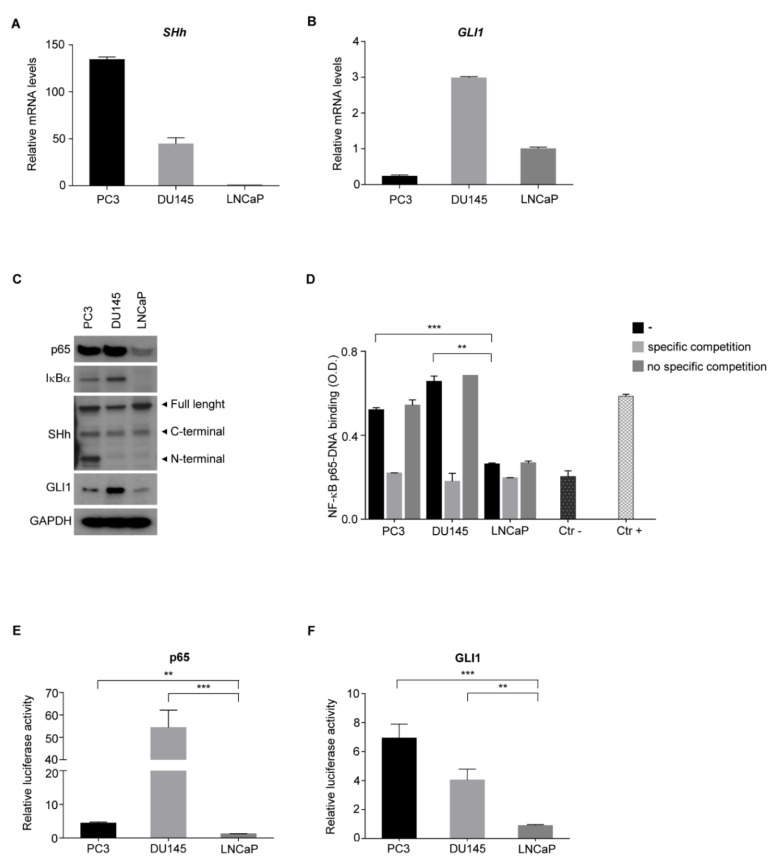
NF-κB promotes the SHh pathway in AI PCa cell lines. (**A**,**B**) Real-time PCR showing basal level of *SHh* (**A**) and *GLI1* (**B**) in AI (PC3, DU145) and AD (LNCaP) cell lines. (**C**) Western blots showing the basal level of p65, IκBα, SHh and GLI1 proteins in AI and AD cell lines. GAPDH is shown as the loading control. (**D**) TransAM assay in AI (PC3, DU145) and AD (LNCaP) cell lines. (**E**,**F**) Dual luciferase reporter assay for p65 (**E**) and GLI1 (**F**) in AI and AD cell lines used in (**A**). Luciferase activity was normalized to Renilla activity. All experiments were performed in triplicate. Values are shown as mean ± S.D. Unpaired Student *t*-test or Mann–Whitney U-test were used for statistical analysis. ** *p* < 0.01; *** *p* < 0.001.

To shed light on the molecular mechanisms underlying the crosstalk between NF-κB(p65) and SHh pathways in advanced PCa, androgen-independent (AI: PC3 and DU145) and androgen-dependent (AD: LNCaP) cell lines were analyzed. qRT-PCR analysis showed an increased expression of *SHh* in PC3 and DU145 compared to LNCaP cells (Figure 3A). The highest *GLI1* expression was observed in the DU145 cell line (Figure 3B).

To gain more insight about the NF-κB(p65)-SHh-GLI1 axis in PCa, we analyzed both NF-κB(p65) and SHh-GLI1 pathways in Western blot. As shown, IκBα protein, a marker of NF-κB activation [42], was present only in AI cell lines, while no expression was observed in AD cells (Figure 3C and Figure S3A–F). Surprisingly, all three SHh isoforms were expressed in the PC3 cell line, while DU145 and LNCaP did not show the N-terminal active isoform of SHh [43]. GLI1 was mainly expressed in DU145 cells (Figure 3C and Figure S3A–F). Indeed, differently from qRT-PCR data, GLI1 protein level was slightly higher in PC3 compared to LNCaP cells (Figure 3C and Figure S3F), probably due to post-translational modifications which impact on GLI1 protein stability [44]. To further assess the NF-κB(p65) and GLI1 transcriptional activity in AI versus AD prostate cell lines, the NF-κB(p65) DNA binding assay (Figure 3D) and luciferase reporter assay specific for NF-κB(p65) (Figure 3E) and GLI1 (Figure 3F) were performed. As expected, the binding of NF-κB(p65) on the κB-consensus binding site (5′-GGGRNWYYCC-3′) was observed by using nuclear extract of PC3 and DU145 cells lines, while no significant binding was detected by using nuclear extract of LNCaP (Figure 3D). Furthermore, the luciferase reporter assay demonstrated high NF-κB(p65) and GLI1 transcriptional activity in both PC3 and DU145 AI cells (Figure 3E,F). Notably, the strong GLI1 transcriptional activity seen in PC3 cells suggests that post-transcriptional modification might occur and enhance DNA binding and transcriptional activity, as already shown in other tumors [45]. In addition, although it is widely accepted that the NF-κB(p65)-SHh-GLI1 axis is active in advanced PCa, DU145 showed NF-κB(p65) and GLI1 activation despite the absence of the active form of SHh (N-terminal). Therefore, in this cell line, GLI1 is activated in a SHh-independent manner, as seen in other tumors [46].

### 3.4. AI PCa Cells Rely on NF-κB/GLI1 Activity for Survival

**Figure 4:**
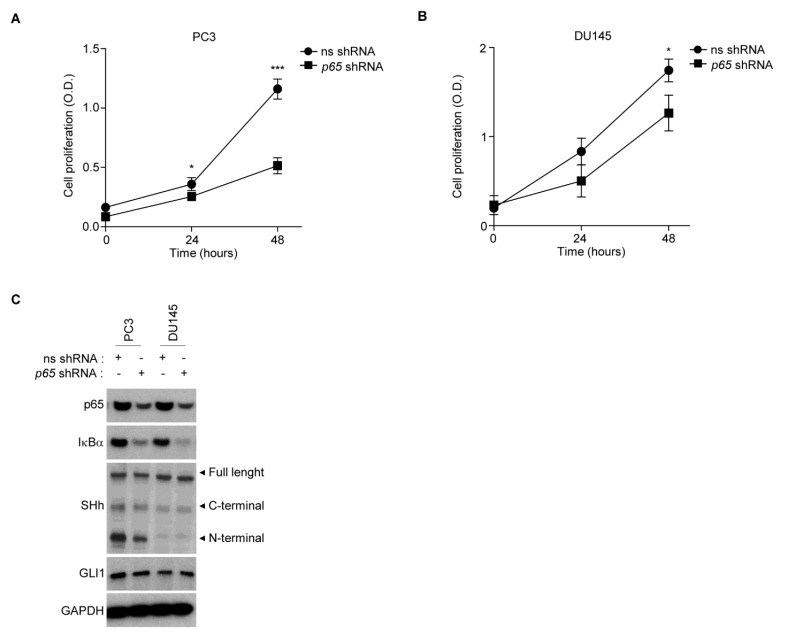
PCa cells rely on NF-κB activity for survival. (**A**,**B**) The MTS assay showing the basal proliferation levels of PC3 (**B**) and DU145 (**C**) cell lines following lentiviral infection of non-specific (sh-ns) and *p65*-specific (sh-*p65*) shRNAs, at the indicated time points. (**C**) Western blot analysis showing the protein levels of p65, IκBα, SHh and GLI1 proteins in the same cell lines used in (**A**,**B**). GAPDH is shown as loading control. (**A**,**B**) Values are shown as mean ± S.D. Unpaired Student *t*-test or Mann–Whitney U-test were used for statistical analysis. * *p* < 0.05; *** *p* < 0.001. All experiments were performed in triplicate.

To functionally evaluate the proliferation rate in our panel of PCa cell lines, we conducted an MTS proliferation analysis (Figure S4A). AI cells showed a higher proliferation rate compared to AD cells, in accordance with the literature [47]. We hypothesized that the NF-κB(p65)/SHh/GLI1 axis could be, at least in part, responsible for the phenotype of AI cell lines. Thus, to elucidate whether NF-κB(p65) signaling contributed to the increased proliferation rate of AI cell lines, we transfected these cells with a specific (sh-*p65*) or non-specific (sh-ns) short harpin using lentiviral particles. We showed that the silencing of NF-κB(p65) significantly reduced proliferation in both PC3 and DU145 (Figure 4A,B), confirming the NF-κB-dependent survival in both AI cell lines.

We further evaluated how NF-κB genetic blockade affected SHh signaling in these cell lines. According to the literature [29], SHh levels were reduced by inhibition of p65 in PC3 cells, and slight downregulation of GLI1, as expected, was observed as well (Figure 4C and Figure S4B–G). On the contrary, no effects were observed in DU145 following p65 silencing, demonstrating that NF-κB(p65) was not responsible for GLI1 activation in these cells.

### 3.5. Pharmacological Inhibition of NF-κB and GLI1 Reduces Cell Survival

**Figure 5:**
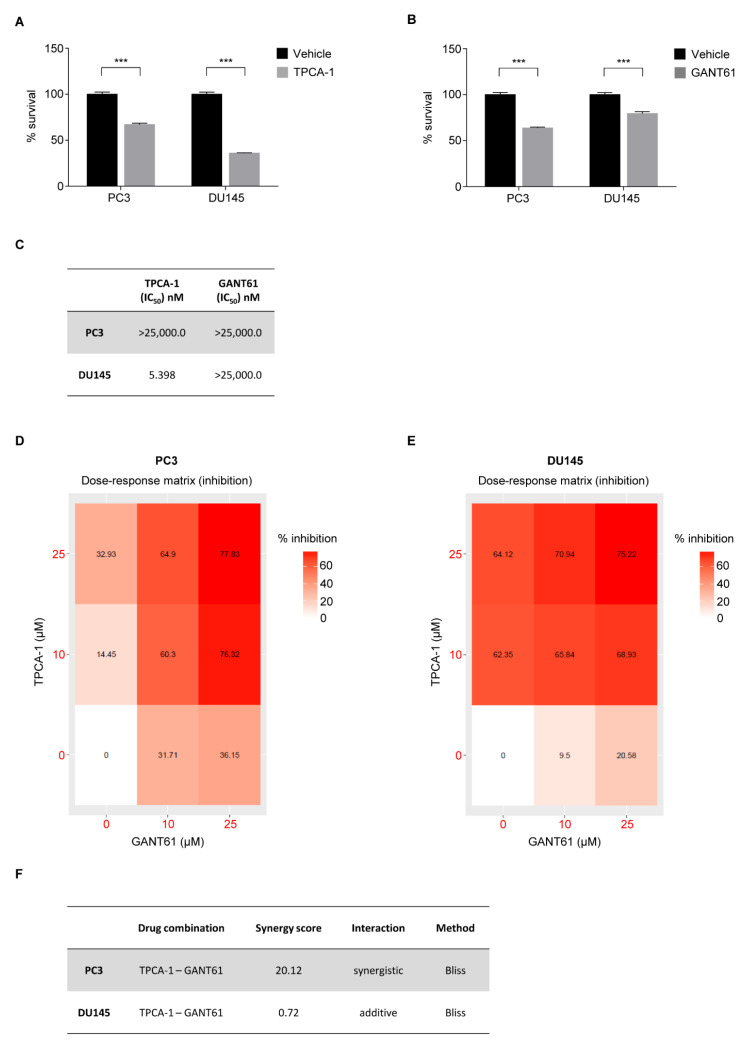
Inhibition of NF-κB and GLI1 kills AI cell lines. (**A**,**B**) CellTiter-Glo assays showing the viability of PC3 and DU145 cell lines following 72 h of treatment with TPCA-1 (**A**) and GANT61 (**B**) or vehicle. (**C**) Shown are IC_50_ values of TPCA-1 and GANT61 at 72 h for the experiment reported in (**A**,**B**). (**D**,**E**) Heatmap showing the dose–response matrix (% inhibition) for TPCA-1 and GANT61 of PC3 (**D**) and DU145 (**E**) cell lines after 72 h of treatment. (**F**) Shown is the synergy score of TPCA-1 and GANT61 for the experiments shown in (**D**,**E**). (**A**,**B**) Values are shown as mean ± S.D. Unpaired Student *t*-test or Mann–Whitney U-test were used for statistical analysis. *** *p* < 0.001. All experiments were performed in triplicate. (**C**,**F**) PC3 (grey) DU145 (white).

To validate the role of NF-κB(p65) in sustaining AI PCa proliferation, we treated PC3 and DU145 cell lines with the specific IKK (IκB kinase) inhibitor TPCA-1 (Figure 5A). Pharmacological blockade of NF-κB(p65) signaling with TPCA-1 showed a significant reduction of cell viability in both AI cell lines, with a higher dose-dependent reduction in DU145 compared to PC3 cells (Figure 5A,C and Figure S5A).

To assess how AI cell line proliferation rates could be impacted from GLI1, PC3 and DU145 cell lines were treated with the GLI1 inhibitor GANT61. GANT61 treatment demonstrated a significant efficacy in reducing cell survival in both cell lines. Surprisingly PC3 cells were reported to be more sensitive to GANT61 than DU145 (IC_50_ = 5.398 nM) (Figure 5B,C and Figure S5B).

Given that NF-κB and SHh-GLI1 pathways sustain cell proliferation in advanced PCa and based on our findings demonstrating the existence of an active crosstalk between these two pathways, we hypothesized that the co-inhibition of NF-κB and SHh-GLI1 pathways could be a new potential therapeutic approach to treat PCa with constitutive activation of the NF-κB/GLI1 axis. To this end, we treated AI cell lines with a combination of TPCA-1 and GANT61. A dose-dependent inhibition (% inhibition) was observed at 72 h post treatment in both cell lines and the drug response analysis demonstrated a synergistic activity of TPCA-1 in combination with GANT61 in killing PC3 (synergy score of 20.12), while an additive interaction was observed against DU145 (synergy score of 0.72) (Figure 5D–F and Figure S6).

Altogether, our data demonstrated that AI cell survival relies on NF-κB and GLI1 activity. Therefore, targeting the existing crosstalk between NF-κB and GLI1, despite SHh involvement, could be a potential successful strategy to treat advanced PCa that is characterized by activation of NF-κB and GLI1 signaling.

## 4. Discussion

Despite the advances that have been made in the treatment of PCa in recent years, mortality for men diagnosed with metastatic PCa remains significantly high [48]. Whereas treatment outcomes for localized PCa have been favorable, metastatic PCa remains incurable and extensive research is on-going to clarify the molecular basis of disease progression. Our study demonstrated the existence of a positive correlation between the activation of NF-κB(p65) and Sonic Hedgehog pathways in PCa, confirming the key role of this axis in PCa [20,31,46]. Overexpression of NF-κB(p65), SHh and GLI1 was observed in aggressive PCa tissue cores featuring high Gleason score and advanced clinical stage and was correlated with worse PFI, corroborating the clinical relevance of the NF-κB(p65)-SHh-GLI1 axis in advanced PCa. Thus, the combination of this RNA-based gene signature and IHC findings for p65, SHh and GLI1 may predict the progression of PCa to advanced stages, directing clinicians to focus on this adverse prognostic profile and to consider alternative therapies.

Although the involvement of both NF-κB and SHh signaling in PCa has already been established and SHh-GLI1 has been reported to repress androgen receptors [28,49,50], little is known about the interplay between these two pathways. To this end, we observed a constitutive activation of NF-κB(p65) and SHh-GLI1 pathways within the highly proliferative cell lines PC3 and DU145. Surprisingly, GLI1 activation seemed to be SHh-dependent only in PC3 cells, while in DU145 GLI1 expression depends neither on SHh or NF-κB(p65). This non-canonical activation of GLI1 has been demonstrated in several tumors and could involve several routes such as RAS-RAF-MEK, PI3K/AKT/mTOR and PKC [28,31,32,46,51,52], but the characterization of the precise molecular mechanism driving SHh/NF-κB(p65)-independent GLI1 activation is beyond the scope of this work.

Further, we demonstrated the potential therapeutic use of NF-κB(p65) and GLI1 inhibitors in treating two cell lines widely used as in vitro models of advanced prostate cancer. The two pathway-specific drugs utilized in our study, TPCA-1 and GANT61, showed a synergic effect when combined in PC3 cells, while an additive response was observed in DU145 [53,54]. While these compounds have already been used as standalone treatments in several preclinical cancer models, no clinical studies are ongoing due to their pharmacological unsuitability, highlighting the need for developing novel drugs that can be used in clinical settings [55,56,57,58]. Recently, key questions about intrapatient, intratumor and interpatient heterogeneity have been solved due to the deeper molecular characterization of PCa, thus supporting the need to develop and implement new prognostic factors and combination therapies. In this context, our data provide new useful insights for the PCa-prognosis and prospectively for define a new route for the treatment of advanced PCa. While many key questions remain unanswered and deeper investigation regarding the translational applications of this crosstalk is needed, this study represents a new step forward for the understanding of NF-κB(p65)-SHh-GLI1 interplay in PCa.

## Data Availability

Expression and survival data for the analysis of The Cancer Genome Atlas (TCGA) Prostatic Cancer (PRAD) program were from the Pan-Cancer Atlas publication [39] and downloaded from the Genomic Data Common repository (https://gdc.cancer.gov/node/905/, accessed on 1 April 2022).

## Supplementary Materials

The following supporting information can be downloaded at: https://www.mdpi.com/article/10.3390/cells11132118/s1. Figure S1. Table summarizing the key clinical data of PCa patients from the TCGA_PRAD data set. Figure S2. (A) Overview of PCa TMAs stained for p65, SHh, GLI1. (B) Representative images of IHC staining for p65, SHh, GLI1 in tumor sections core. (C–E) IHC analysis showing the percentage of positive area for p65 (C), SHh (D) and GLI1 (E) in PCa tissue samples from (A) stratified by normal (N) and tumor (T). (C–E) Unpaired Student *t*-tests were used for statistical analysis. * *p* < 0.05; ** *p* < 0.01. Scale and magnifications are shown. Figure S3. (A–F) Densitometry analysis for p65 (A), IκBα (B), SHh (Full length) (C), SHh (C-terminal) (D), SHh (N-terminal) (E), and GLI1 (F) performed on Western blot shown in Figure 3C. Figure S4. (A) MTS assay showing the basal proliferation levels of AI and AD cell lines at the indicated time points. (B–G) Densitometry analysis for p65 (B), IκBα (C), SHh (Full length) (D), SHh (C-terminal) (E), SHh (N-terminal) (F), and GLI1 (G) performed on Western blot shown in Figure 4C. Figure S5 (A,B) CellTiter-Glo assays showing the viability of PC3 and DU145 cell lines after 72 h of treatment with the indicated concentrations of TPCA-1 (A) and GANT61 (B). Values are mean ± S.D. Figure S6 (A,B) 2D Synergy map for PC3 (A) and DU145 (B) cell lines after 72 h of treatment with the combination of TPCA-1 and GANT61 at the indicated concentrations. Red and green colors highlight synergistic and antagonistic dose regions, respectively.
