# Supplementary material for: Elevated NF-κB/SHh/GLI1 Signature Denotes a Worse Prognosis and Represent a Novel Potential Therapeutic Target in Advanced Prostate Cancer
Source: Cells. 2022 Jul 5;11(13):2118. doi: 10.3390/cells11132118 (PMC9266159; doi:10.3390/cells11132118)
Supplement: Supplementary file 1 [file cells-11-02118-s001.zip › cells-1779625-supplementary.pdf]

TCGA_PRAD Patient characteristics	
Total Prostate Adenocarcinoma Acinar Type	482
Age Mean	60.99 ± 6.85
Alive	473
Dead	9
**PSA**	
NA	57
>8	18
**Radiation Therapy**	
NO	380
YES	57
NA	45
**Pathological T**	
T2a	13
T2b	10
T2c	162
T3a	152
T3b	128
T4	10
NA	7
**Gleason Score**	
6	45
7	245
8	58
9	130
10	4

Figure S1. Table summarizing the key clinical data of PCa patients from the TCGA_PRAD data set.

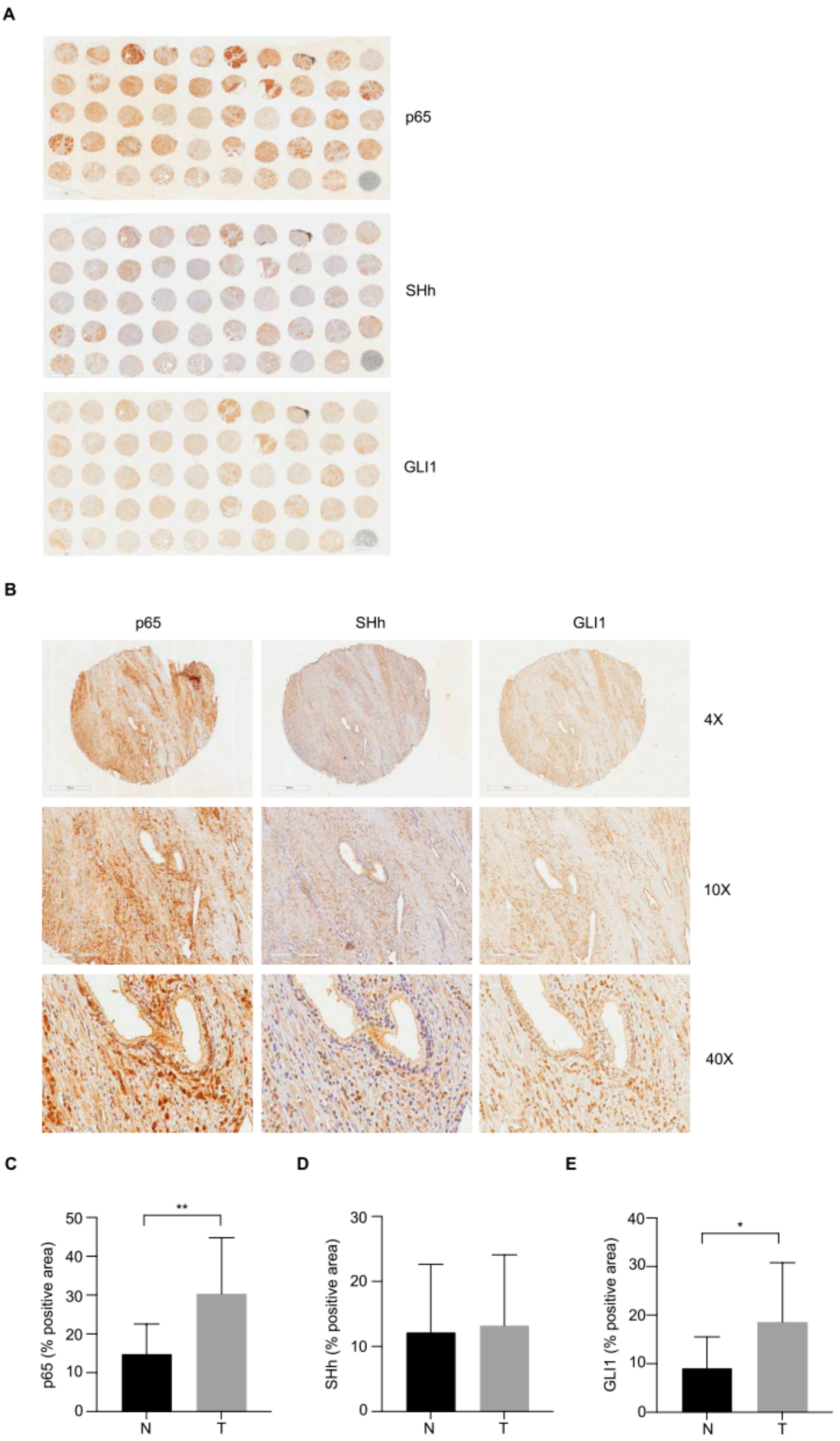

Figure S2. (A) Overview of PCa TMAs stained for p65, SHh, GLI1. (B) Representative images of IHC staining for p65, SHh, GLI1 in tumor sections core. (C–E) IHC analysis showing the percentage of positive area for p65 (C), SHh (D) and GLI1 (E) in PCa tissue samples from (A) stratified by normal (N) and tumor (T). (C–E) Unpaired Student *t*-tests were used for statistical analysis. * $p < 0.05$; ** $p < 0.01$. Scale and magnifications are shown.

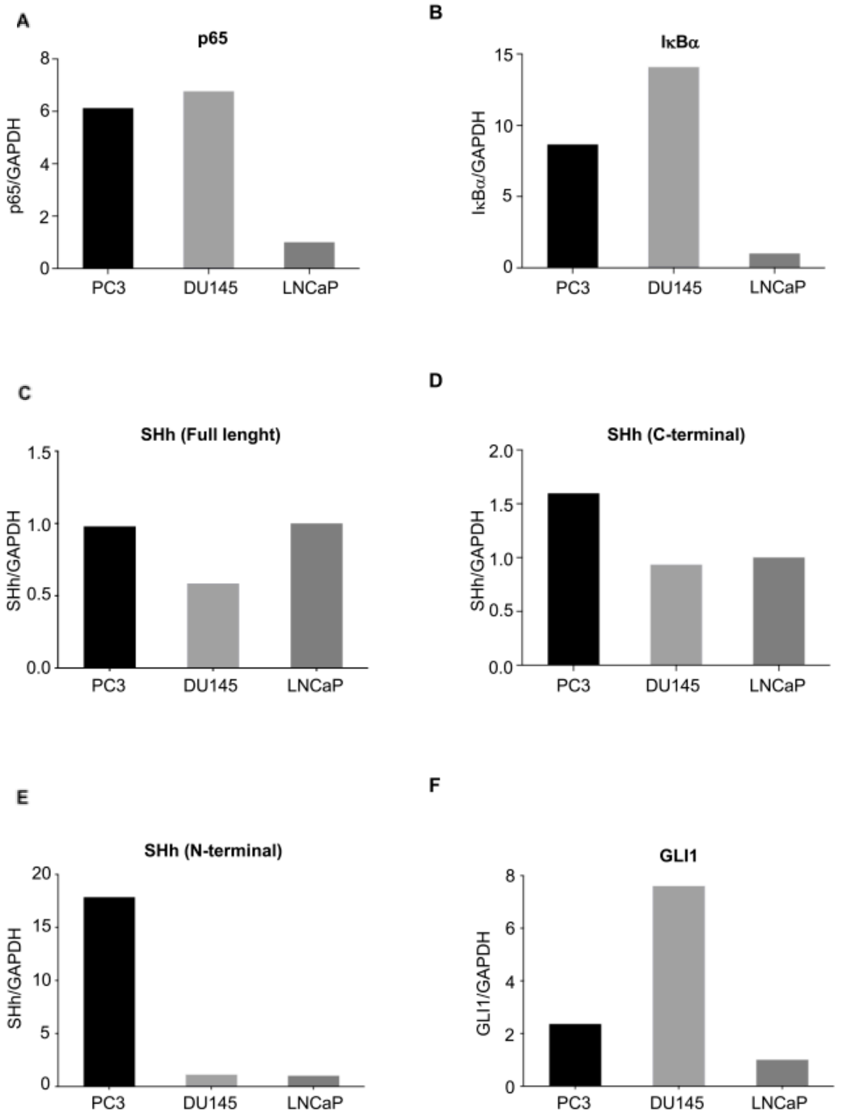

Figure S3. (A–F) Densitometry analysis for p65 (A), IκB*α* (B), SHh (Full length) (C), SHh (C-terminal) (D), SHh (N-terminal) (E), and GLI1 (F) performed on Western blot shown in Figure 3C.

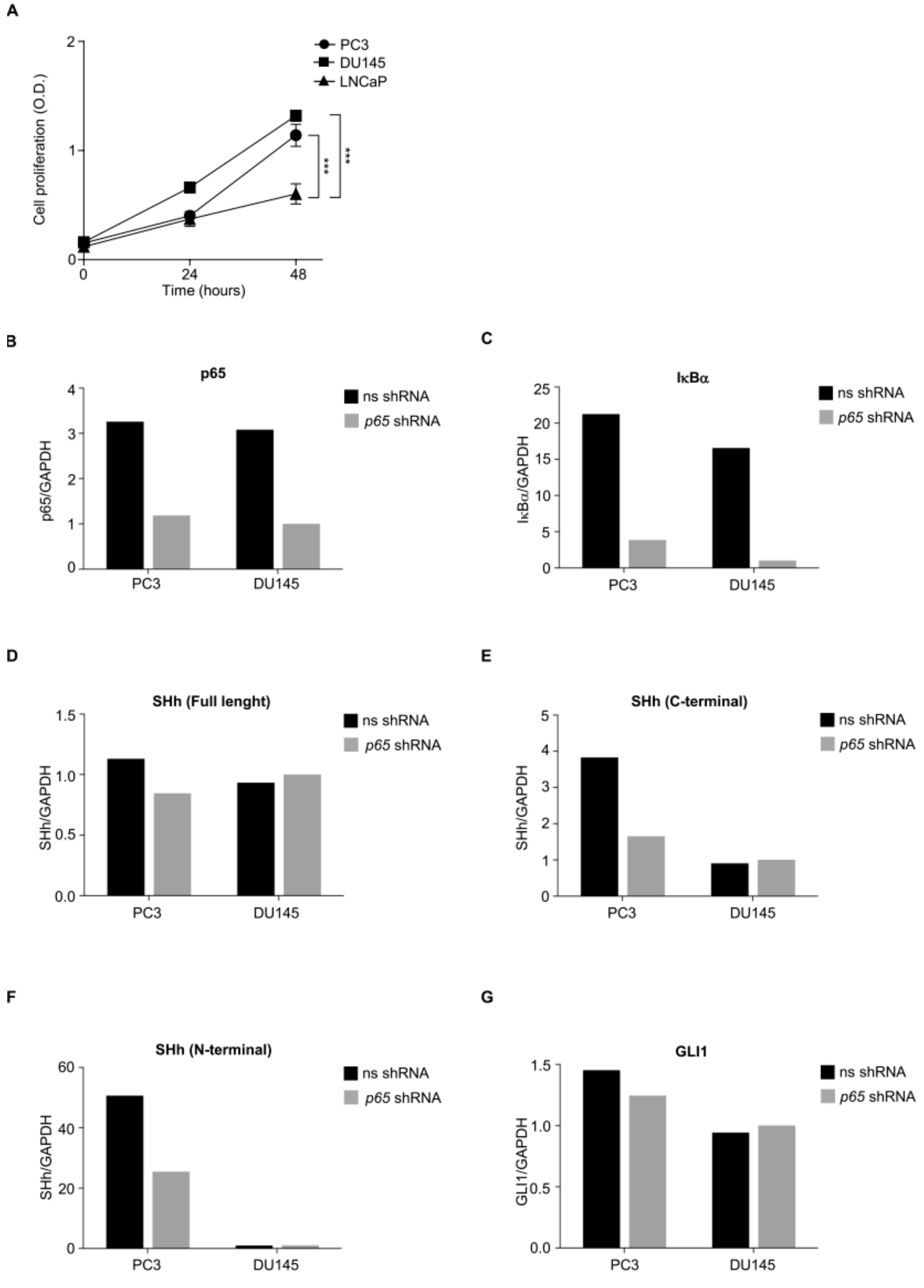

Figure S4. (A) MTS assay showing the basal proliferation levels of AI and AD cell lines at the indicated time points. (B–G) Densitometry analysis for p65 (B), IκBα (C), SHh (Full length) (D), SHh (C-terminal) (E), SHh (N-terminal) (F), and GLI1 (G) performed on Western blot shown in Figure 4C.

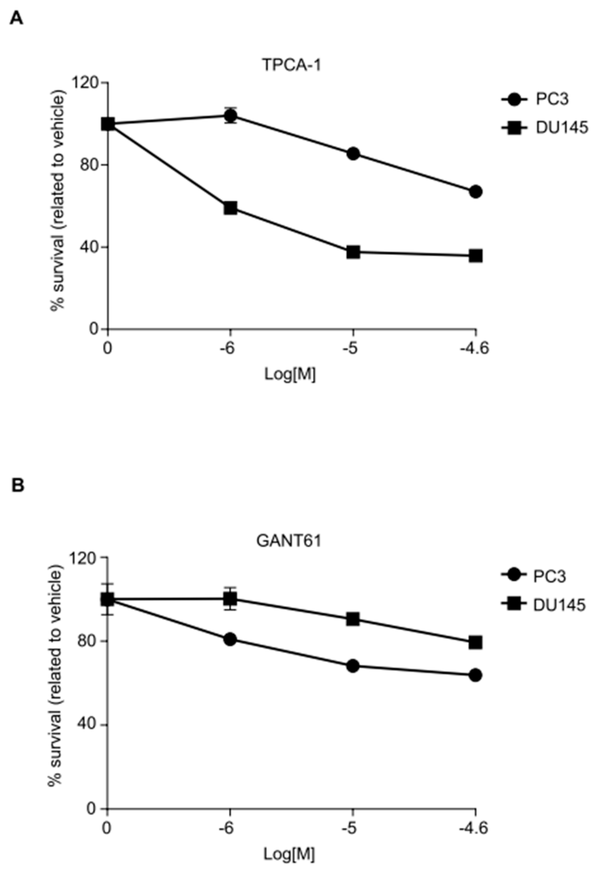

Figure S5. (A-B) CellTiter-Glo assays showing the viability of PC3 and DU145 cell lines after 72 hours of treatment with the indicated concentrations of TPCA-1 (A) and GANT61 (B). Values are mean ± S.D.

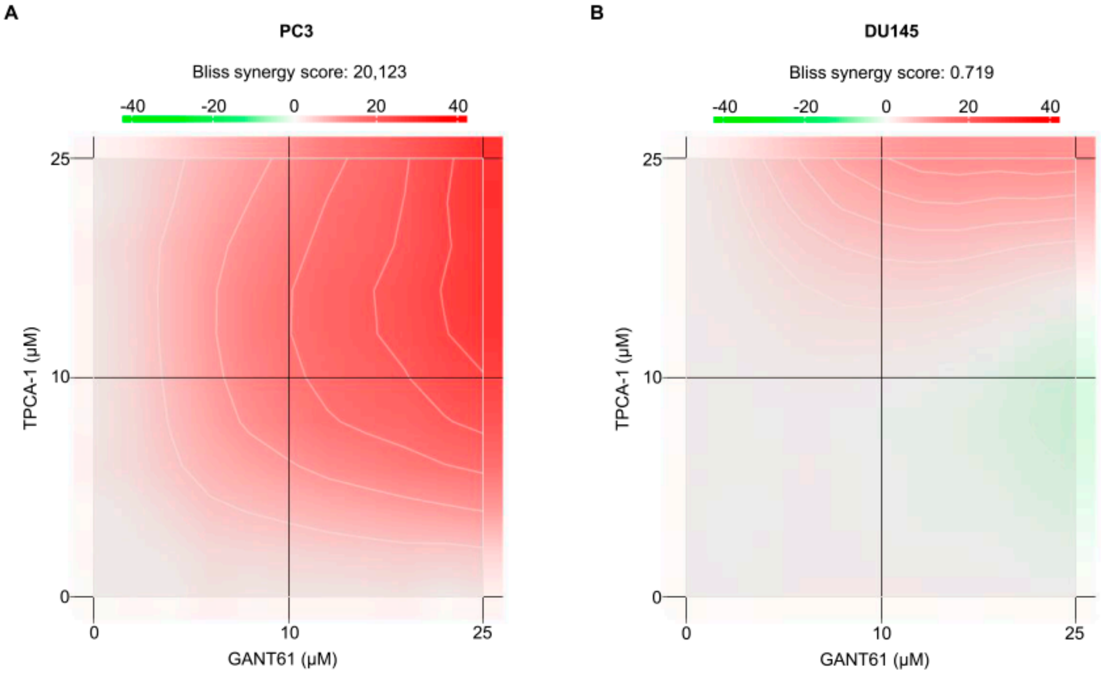

Figure S6. (A,B) 2D Synergy map for PC3 (A) and DU145 (B) cell lines after 72 hours of treatment with the combination of TPCA-1 and GANT61 at the indicated concentrations. Red and green colors highlight synergistic and antagonistic dose regions, respectively.
